# Thiol-based redox control of enzymes involved in the tetrapyrrole biosynthesis pathway in plants

**DOI:** 10.3389/fpls.2013.00371

**Published:** 2013-09-20

**Authors:** Andreas S. Richter, Bernhard Grimm

**Affiliations:** Department of Plant Physiology, Institute of Biology, Mathematisch-Naturwissenschaftliche-Fakultät I, Humboldt-University BerlinBerlin, Germany

**Keywords:** tetrapyrrole biosynthesis, redox regulation, post-translational, plant, ALA synthesis

## Abstract

The last decades of research brought substantial insights into tetrapyrrole biosynthetic pathway in photosynthetic organisms. Almost all genes have been identified and roles of seemingly all essential proteins, leading to the synthesis of heme, siroheme, phytochromobilin, and chlorophyll (Chl), have been characterized. Detailed studies revealed the existence of a complex network of transcriptional and post-translational control mechanisms for maintaining a well-adjusted tetrapyrrole biosynthesis during plant development and adequate responses to environmental changes. Among others one of the known post-translational modifications is regulation of enzyme activities by redox modulators. Thioredoxins and NADPH-dependent thioredoxin reductase C (NTRC) adjust the activity of tetrapyrrole synthesis to the redox status of plastids. Excessive excitation energy of Chls in both photosystems and accumulation of light-absorbing unbound tetrapyrrole intermediates generate reactive oxygen species, which interfere with the plastid redox poise. Recent reports highlight ferredoxin-thioredoxin and NTRC-dependent control of key steps in tetrapyrrole biosynthesis in plants. In this review we introduce the regulatory impact of these reductants on the stability and activity of enzymes involved in 5-aminolevulinic acid synthesis as well as in the Mg-branch of the tetrapyrrole biosynthetic pathway and we propose molecular mechanisms behind this redox control.

## INTRODUCTION

Regulation of the redox status of processes taking place in plastids, including the tetrapyrrole metabolism, by disulfide bond formation and reduction is one of the crucial post-translational control mechanisms to modulate protein activity, folding, and stability. The exploration of thiol-based control of enzymatic steps in tetrapyrrole biosynthesis has just been initiated. We will report about the enzymes which are proposed to be subjected to thiol-based regulation in Sections “Redox Modifications of ALA Synthesis Enzymes” and “Redox Control in the Mg-Branch.” The thiol-reducing systems that control the redox state of enzymes of tetrapyrrole will be introduced in Section “Thiol-Based Redox Regulators of Higher Plants,” while the significant role of post-translational redox control in tetrapyrrole biosynthesis will be discussed in Section “The Need for Post-Translational Redox Regulation in Tetrapyrrole Biosynthesis.” An initial overview of the tetrapyrrole biosynthetic pathway is given in the following chapter.

## THE OVERVIEW OF THE TETRAPYRROLE BIOSYNTHETIC PATHWAY IN PLANTS

Plant tetrapyrrole biosynthesis results in the formation of multiple end products, including chlorophyll (Chl), heme, siroheme, and phytochromobilin. These biomolecules belong to the most abundant macromolecules in the plant kingdom and are formed through a complex metabolic pathway that consists of at least 25 single enzymatic reactions in plastids (Tanaka and Tanaka, 2007; Mochizuki et al., 2010).

**FIGURE 1:**
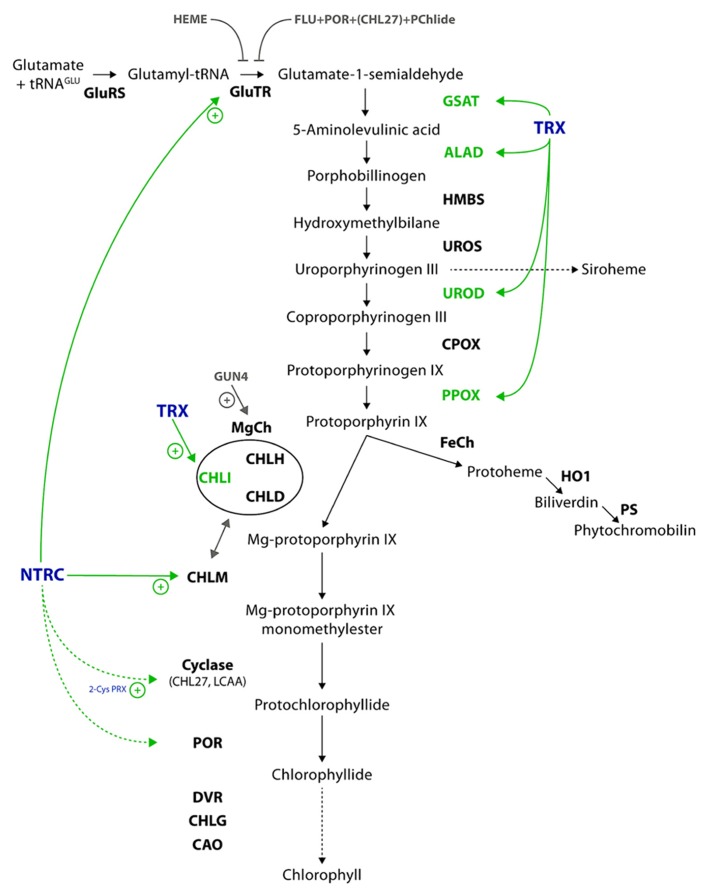
**Scheme of the plant tetrapyrrole biosynthetic pathway.** The coloring highlights the redox regulation and targets of redox regulation in green and the involved enzymes in blue. Further post-translational regulations are indicated in gray, including the proteins involved. Solid lines refer to verified interactions and dotted lines to potential targets of thiol-based regulation. Further details and the abbreviations of the enzymes are given in the text.

The tetrapyrrole metabolism begins with the synthesis of 5-aminolevulinic acid (ALA), the first committed metabolite of the pathway (**Figure 1**). Like most of the prokaryotes, photosynthetic eukaryotes synthesize ALA from glutamate (Glu), while fungi, animals, and α-proteobacteria catalyze the condensation of glycine and succinyl-CoA to ALA by ALA synthase. Plants use Glu which is coupled to glutamyl-transfer RNA (tRNA^Glu^) by glutamyl-tRNA synthase (GluRS). One of the main regulatory enzymes of tetrapyrrole biosynthesis is glutamyl-tRNA reductase (GluTR), which reduces the activated glutamyl-tRNA to Glu-1-semialdehyde (GSA). GSA aminotransferase (GSAT) catalyzes an intramolecular transfer of an amino group of GSA to produce ALA. ALA synthesis is the rate limiting step of the entire pathway and precisely controlled at the transcriptional and post-translational levels by multiple factors (Mochizuki et al., 2010; Tanaka et al., 2011).

In continuation two ALA molecules are merged by ALA dehydratase (ALAD) to form the first monopyrrole porphobilinogen. Hydroxymethylbilane synthase (HMBS) consecutively condenses four molecules of porphobilinogen to its cofactor dipyrromethane, before the final product, the linear tetrapyrrole hydroxymethylbilane (HMB), is released. HMB is converted to uroporphyrinogen III (UroIII) by UroIII synthase (UROS). Siroheme is formed in a three-step reaction from UroIII (not specified in **Figure 1**), while in the main branch of tetrapyrrole biosynthesis UroIII decarboxylase (UROD) and coproporphyrinogen III oxidase (CPOX) catalyze in two sequential reactions the formation of protoporphyrinogen IX (Protogen). Protogen oxidase I (PPOX) catalyzes the removal of six electrons of Protogen to form protoporphyrin IX (Proto). At this point, the tetrapyrrole metabolism branches into two distinct pathways, the Fe- and Mg-branch. Whereas the Fe branch leads to the formation of heme and its derivates, the catalytic reactions of the Mg-branch result in Chl formation. Within the Fe branch ferrochelatase (FeCh) catalyzes the insertion of ferrous ion (Fe^2^^+^) into Proto. Activities of heme oxygenase (HO) and phytochromobilin synthase (PS) convert Proto into biliverdin IX and phytochromobilin, the chromophore of phytochromes, respectively.

The insertion of an Mg^2^^+^ ion into the Proto macrocycle by magnesium chelatase (MgCh) is a highly energy-consuming step. MgCh consists of the three subunits CHLI, CHLD, and CHLH. CHLI and CHLD form each a hexameric ring that interacts with the catalytically active CHLH subunit. CHLI possesses ATPase activity (Jensen et al., 1999), while CHLH catalyzes the Mg^2^^+^insertion into Proto. This assembly of CHLH with the CHLI/CHLD double ring structure is highly important for the ATP-driven chelation reaction (Hansson and Kannangara, 1997; Jensen et al., 1999). In addition the *in vitro* and *in vivo* activity of MgCh is stimulated by the regulator protein GUN4, which was originally identified in a screen of mutants with impaired plastid to nucleus signaling (Susek et al., 1993; Larkin et al., 2003; Peter and Grimm, 2009). The product of the chelation reaction is Mg protoporphyrin IX (MgP) which is methylated through the action of MgP methyltransferase (CHLM). Thereby, a methyl group from *S*-adenosyl methionine is transferred to MgP to form MgP monomethylester (MgPME). Subsequently, the aerobic MgPME oxidative cyclase (herein after referred to as cyclase) catalyses the closure of a fifth ring which is specific for the chlorine-type products of the pathway. The cyclase is assumed to consist of three subunits (Rzeznicka et al., 2005), from which CHL27 (Tottey et al., 2003) and LCAA/YCF54 have been already identified and are considered to be essential subunits for the cyclase activity (Albus et al., 2012).

In angiosperms the strictly light-dependent protochlorophyllide oxidoreductase (POR) reduces the D ring of 3,8-divinyl-protochlorophyllide (DV-Pchlide) to 3,8-divinyl-chlorophyllide (DV-Chlide). Gymnosperms, algae, and cyanobacteria contain two structurally distinct PORs, which catalyze either light-dependently or light-independently Chlide formation. The 8-vinyl group of 3,8-DV-Chlide is then reduced by the DV-reductase (DVR) to form monovinyl Chlide. The DVR also catalyzes the DV reduction of other metabolites of the Mg-branch. In the following step, Chlide is esterified at the 17-propionate group of ring D with phytyl pyrophosphate by Chl synthase (CHLG) to form Chl a. Chl(ide) a oxygenase (CAO) catalyses Chl b formation at the end of the Mg-branch of tetrapyrrole biosynthesis (Tanaka and Tanaka, 2011).

## THIOL-BASED REDOX REGULATORS OF HIGHER PLANTS

Regulation of the thiol–disulfide exchange reactions in metabolic processes is a decisive post-translational modification of proteins that affects their catalytic activity, folding, and turnover. The thiol-reducing system that controls the redox state of enzymes involved in tetrapyrrole biosynthesis is driven either by light and the ferredoxin-thioredoxin-dependent or the NADPH-thioredoxin-dependent pathway. In the first pathway the electrons flow through thiol–disulfide exchanges from ferredoxin to thioredoxin. This reaction is catalyzed by ferredoxin-thioredoxin reductase. In its reduced state, thioredoxin transfers electrons to the respective target proteins. The reducing state is supported by photosynthetic activity and allows thereby the functional separation of day–night activities. Consequently, in darkness the thiol groups of target proteins can be oxidized by oxygen, reactive oxygen species (ROS), oxidized thioredoxin, and glutathione.

In most cases a light-dependent reduction of enzymes correlates with their enhanced activity. Glucose 6-phosphate dehydrogenase, the entry enzyme into the oxidative pentose phosphate cycle, is an exception, and is inactivated by thiol-reducing activities. Initially, two chloroplastic thioredoxins, the f and m type, were reported as the light-dependent post-translational regulators of metabolic activity. At present, several other types of thioredoxins have been reported, which are classified according to their structure, target proteins, and localization (Chibani et al., 2010). The high multitude of thioredoxin-like proteins in photosynthetic organisms is surprising and challenging in terms of the identification of their specific functions by reverse genetic approaches. Functional redundancy of these thioredoxins in terms of their capacity to reduce thiol groups of the same target proteins most likely explains the repeatedly observed lack of phenotypical effects when single thioredoxin-encoding genes were inactivated. Using thioredoxin-affinity chromatography several proteins of tetrapyrrole biosynthesis have been identified as potential targets of thioredoxin: GSAT, UROD, ALAD, and PPOX (**Figure 1**; Balmer et al., 2003; Marchand et al., 2006). Pheophorbide a oxygenase and red Chl catabolite reductase are two enzymes of the Chl catabolic pathway and were also identified in proteomic studies as thioredoxin targets (Bartsch et al., 2008).

Apart from the ferredoxin-thioredoxin-dependent redox system, a NADPH-dependent thioredoxin reductase (NTR) contributes to the control of tetrapyrrole biosynthesis. Two out of three *Arabidopsis* NTR isoforms, NTRA and NTRB, are dual-targeted to the cytoplasm and mitochondria (Reichheld et al., 2005). The NTRC isoform is localized in plastids and, exceptionally, consists of a NTR module and the thioredoxin module (Serrato et al., 2004). NADPH donates the electrons, which are transferred through FAD to an internal disulfide bond. Following the electron transfer, the reduced cysteines of NTRC serve for the reduction of the thioredoxin domain. NTRC-dependent redox regulation can also occur during dark periods using NADPH generated, e.g., in the oxidative pentose phosphate pathway. Therefore, redox regulation of target enzymes by NTRC can occur independent from photosynthetic activities. It was previously shown that the combined action of NTRC and 2-cysteine-peroxiredoxin (2-Cys PRX) protects the MgPMME cyclase in *in vitro* enzyme assays (Stenbaek et al., 2008). Apart from the initial observations of a NTRC-dependent sensitivity of the cyclase reaction, NTRC deficiency had a strong impact on various proteins in tetrapyrrole biosynthesis. Western blot analysis of the tetrapyrrole biosynthesis enzymatic proteins in a *ntrc* mutant of *A. thaliana* revealed reduced content of GluTR, POR, and CHLM (Richter et al., 2013; see also Redox Modifications of ALA Synthesis Enzymes and Redox Control in the Mg-Branch).

It has been found in recent studies that glutathione participates in a post-translational modification, named glutathionylation, consisting of the reversible formation of mixed disulfides between a thiol group of the target protein and the thiol of glutathione (Dixon et al., 2005; Rouhier et al., 2008; Zaffagnini et al., 2012). This post-translational modification is generally promoted by ROS such as hydrogen peroxide and protects cysteine residues from irreversible oxidation (see also The Need for Post-Translational Redox Regulation in Tetrapyrrole Biosynthesis). But it has also been shown that enzymatic activity of glutathionylated proteins was altered. While glutathionylation can occur via non-enzymatic mechanisms *in vivo*, the reverse reaction (namely deglutathionylation) is catalyzed by glutaredoxins. These are small oxidoreductases also belonging to the thioredoxin superfamily.

Using radioactively labeled glutathione, a proteomic approach in *Chlamydomonas* revealed 25 target proteins, mainly in the chloroplast. Among the identified proteins, the CHLI subunit of MgCh was shown to be glutathionylated (Michelet et al., 2008). Additional targets in tetrapyrrole metabolism cannot be excluded and glutathione function has to be explored to clarify potential roles in redox regulation, protection, and signaling through glutathionylation and deglutathionylation of specific target proteins.

Apart from post-translational redox modifications, some enzymes of tetrapyrrole biosynthesis contain metal cofactors or iron–sulfur clusters as active redox center in their catalytic centers. These cofactors function in electron transfer reactions to tetrapyrrole substrates and are served with electrons from ferredoxin. Although these reactions do not belong to thiol-dependent redox reactions, these electron distributions are briefly mentioned at the end of this chapter.

Ferredoxin is a [2Fe–2S] cluster containing enzyme that divert electrons to NADP^+^, but also to other redox-mediating components and is therefore essential in the metabolic reactions including Chl biosynthesis (Hanke and Mulo, 2013). The final enzyme of Chl synthesis, CAO, contains a Rieske-type iron–sulfur cluster[2Fe–2S] and receives electrons from ferredoxin. A structurally related enzyme of Chl degradation, pheophorbide a oxygenase also catalyses the electron transfer via the [2Fe–2S] cluster. Thereby, ring opening of pheophorbide a generates the product red Chl catabolite. Sirohydrochlorin FeCh which belongs to the siroheme biosynthesis branch, as well as Chl b reductase and 7-hydroxymethyl Chl a reductase, two enzymes of the Chl cycle, are all ferredoxin-dependent enzymes (Tanaka and Tanaka, 2011).

Ferredoxin participates also in reactions of heme degradation and synthesis of phytochromobilin, the chromophore of phytochrome (**Figure 1**). Protoheme degradation is initiated by a ring opening reaction catalyzed by HO. This enzyme transfers electrons from ferredoxin to heme and catalyses the formation of biliverdin IX. The successive enzyme, PS, reduces biliverdin IX in a ferredoxin-dependent step to phytochromobilin.

In summary, there are first experimental evidences for physical interactions of potential redox regulated enzymes with redox regulators arguing for a control of the tetrapyrrole biosynthetic pathway in response to the redox poise of chloroplasts. But, despite these experimental hints the need for a specific redox regulation of each enzyme has to be scrutinized in future in its physiological environment.

## THE NEED FOR POST-TRANSLATIONAL REDOX REGULATION IN TETRAPYRROLE BIOSYNTHESIS

**FIGURE 2:**
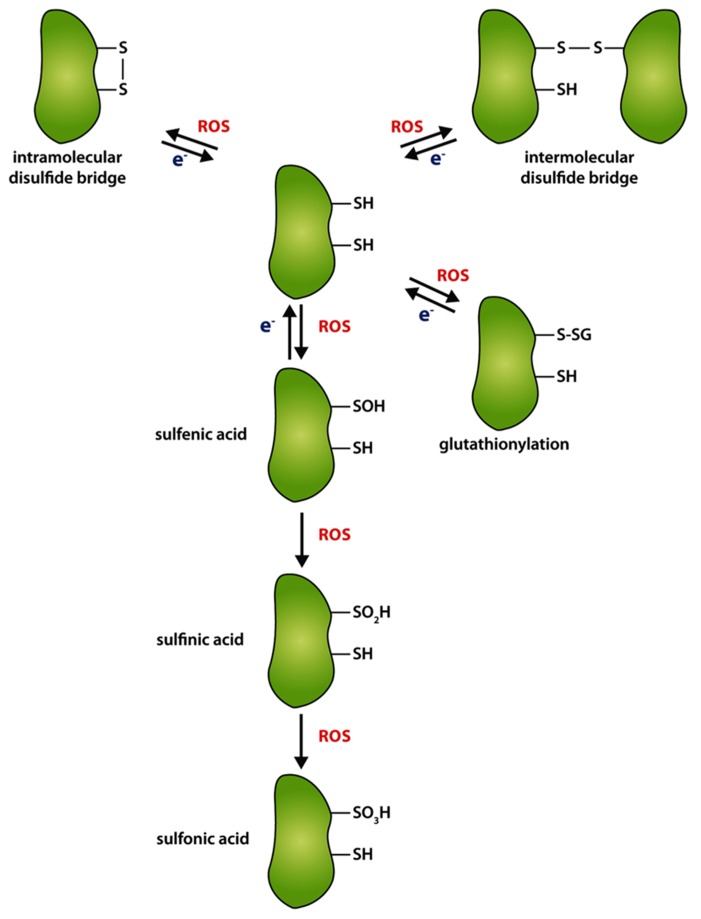
**Differential oxidation of thiol groups of cysteine residues.** Oxidation of cysteine residues by reactive oxygen species (ROS) can lead to the formation of intramolecular (upper left corner) or intermolecular disulfide bridge (upper right corner), respectively. In the absence of an appropriate interaction partner or a second cysteine for disulfide bridge formation, cysteines can be stepwise oxidized to sulfenic (–SOH), sulfinic (–SO_2_H), or sulfonic acid (–SO_3_H). Mixed disulfides can be formed with glutathione (GS). Re-reduction (e^-^) of oxidized cysteines is performed by target-specific enzymes, like thioredoxin, NTRC, or GRX.

Cysteine residues are the main redox active components in proteins. Their thiol groups can be reversibly oxidized to stabile intra- or intermolecular disulfide bonds (**Figure 2**). The thiol–disulfide exchanges occur despite the reducing environment in intracellular compartments, such as the cytoplasm, plastidic stroma, or mitochondrial matrix, and in oxidative environment of the extracellular space, including the apoplasm, the vacuole and the endoplasmic reticulum. The thiol group modifications of cysteines are particularly susceptible to oxidation by ROS (*S*-sulfenation) and nitric oxide (NO, *S*-nitrosylation). Dependent on subcellular restricted oxidative conditions, stable intra- and intermolecular disulfide bonds of proteins or mixed disulfide bonds with reduced glutathione (GSH) as well as less stable sulfenic acid side chains can be formed. These thiol switches (thiol–disulfide exchange reactions) lead to structural alterations of proteins indicating that cysteine residues can be critical for protein function and structure.

The need of thiol-based regulation of tetrapyrrole biosynthetic pathway is explained by the regulatory interdependency of a balanced metabolic flow during rapid environmental changes and the high risk of accumulating photosensitizing tetrapyrrole metabolites. One important source of oxidation is the innate production of singlet oxygen (^1^O_2_) in tetrapyrrole biosynthesis, when light-absorbing tetrapyrrole metabolites accumulate as a consequence of a deregulated pathway. Then, these tetrapyrrolic intermediates act as photosensitizers leading to ^1^O_2_ formation by transfer of excitation energy from excited triplet-state of these metabolites to the triplet-state of molecular oxygen (^3^O_2_) upon illumination. Another type of ROS production can occur during photosynthesis, when electrons of the linear photosynthetic transfer chain are incurred from oxygen resulting in the formation of superoxide radicals ($O_{2}^{•-}$; Apel and Hirt, 2004; Asada, 2006). Superoxide is reduced by superoxide dismutase (SOD, in plastids the copper–zinc and iron-dependent SOD) to hydrogen peroxide, which is reduced to water by peroxidases (e.g., ascorbate peroxidase; Apel and Hirt, 2004; Asada, 2006).

The risk of tetrapyrrole-induced ROS formation drastically increases, when the scavenging activity of ROS-detoxifying enzymes, including SOD or peroxidases, is overstrained (Mock and Grimm, 1997). In addition, excess of highly reactive ^1^O_2_ tends to induce lipid peroxidation which can than interfere with various metabolic processes (Vavilin and Vermaas, 2002; Fischer et al., 2013).

The FLU protein of *Arabidopsis *was demonstrated to act as a dark repressor of ALA synthesis. The *flu *mutant accumulates high amounts of non-protein bound Pchlide in the dark (Meskauskiene et al., 2001; Meskauskiene and Apel, 2002; Richter et al., 2010). Upon light irradiation, the excited, free Pchlide in the triplet-state (^3^Pchlide) can react with ^3^O_2_ to produce ROS (Vavilin and Vermaas, 2002; Triantaphylides et al., 2008). It was demonstrated, that in fact all accumulating porphyrin and Mg porphyrin intermediates (and the visible light-absorbing Chl catabolites) enhance ROS production upon light irradiation resulting in phototoxic damage to cellular components and mimicking the effects of photodynamic herbicides (Mock et al., 1995; Kruse et al., 1997; Mock and Grimm, 1997; Lermontova and Grimm, 2000; Peter et al., 2010).

On the whole, the toxicity of all types of ROS depends on their distinct reactivity and signaling activity, half-life and detoxification. Cells are permanently challenged to control and minimize ROS levels in all intracellular compartments by instantaneous detoxification activities, although ROS also act as signaling components for the activation of acclimating antioxidative stress responses. ROS-mediated signaling likely includes reversible modifications of proteins, which ultimately result in adaptation responses. Post-translational modifications of protein thiols, such as thiol–disulfide exchange, sulfenation, glutathionylation, and nitrosylation, could potentially be involved in redox signaling. Singlet oxygen (^1^O_2_) was shown to have an important signaling function in the communication between plastids and the nucleus under certain conditions (Kim et al., 2008,^2012^; Kim and Apel, 2013). However, ^1^O_2_ has a very short half-life in the cell (~200 ns; Gorman and Rodgers, 1992) and can be rapidly quenched by the reaction with water, tocopherols or carotenoids (Asada, 2006; Triantaphylides and Havaux, 2009). Accumulation of other ROS, mainly hydrogen peroxide, can modulate the redox signaling pathway and trigger the response of distinct detoxifying enzymes.

One common feature of all ROS is their non-site-specific reactivity which can result in the oxidation of biomolecules, like pigments, proteins, or lipids. Dependently on an oxidative cellular environment and with increasing ROS levels, thiol groups of cysteine residues become oxidized (Dalle-Donne et al., 2009; Triantaphylides and Havaux, 2009). As mentioned above in the introductory paragraph of this chapter due to its potent accessibility of oxidants, cysteines can form adducts with molecular oxygen to sulfenic (–SOH), or to the irreversible forms sulfinic (–SO_2_H) or sulfonic (–SO_3_H) acids (**Figure 2**). During a less severe oxidative intracellular environment, oxidation of a cysteine thiol group can either lead to the formation of an intra- or intermolecular disulfide bridge between proteins or low-molecular weight mixed disulfides in dependency of the three-dimensional conformation of the protein, the presence of an appropriate interaction partner or other agents with thiol groups (Dalle-Donne et al., 2009). In consequence of the oxidation, active site or regulatory cysteines can influence enzyme activity, conformational integrity, or stability of proteins (Buchanan and Balmer, 2005; Luo et al., 2012; Richter et al., 2013). The re-reduction of disulfide bridges and thus opening of the S–S or reduction of –SOH can be performed by several target-specific redox regulators which transfer electrons to target cysteines.

## REDOX MODIFICATIONS OF ALA SYNTHESIS ENZYMES

Section “The Overview of the Tetrapyrrole Biosynthetic Pathway in Plants” introduced the complete pathway of tetrapyrrole biosynthesis of photosynthetic organisms, including ALA biosynthesis. According to previous reports the two first pathway-specific enzymes, GluTR and GSAT, are potential candidates of thiol-based post-translational regulation (Buchanan and Balmer, 2005; Richter et al., 2013). Thus, it is likely that the initial flux-regulating enzymatic steps are controlled in response to the plastid redox modifications by reversible thiol–disulfide exchange reactions of contributing enzymes causing changes in their activity and conformation (**Figure 1**).

Although the GluTR from the archaeon* Methanopyrus kandleri* was found as a tetrameric enzyme when purified from *E. coli* lysates by size exclusion chromatography (Moser et al., 1999). GluTR crystals revealed a homodimerization of two GluTR molecules (Moser et al., 2001). GluTR homodimerization was confirmed for the *Arabidopsis *protein *in planta *using bi-molecular fluorescence complementation assays (Czarnecki et al., 2011). The archaebacterial GluTR dimer forms a V-shaped structure (Moser et al., 2001) and the two monomers interact with each other at their C-terminal dimerization domain (Schubert et al., 2002). Interestingly, the crystallized dimer of GSAT from *Synechococcus *could be modeled into the V-shaped notch of homodimeric GluTR (Hennig et al., 1997; Schubert et al., 2002). However, the direct *in planta *interaction of GluTR and GSAT has still not been proven by affinity chromatography or protein interaction approaches. In addition the high reactivity of GSA, the interim metabolite between both enzymes, makes substrate channeling likely. The proposed tight interaction between GluTR and GSAT would enable a direct contact of the two catalytic sites to ease substrate channeling of GSA between both enzymes.

**FIGURE 3:**
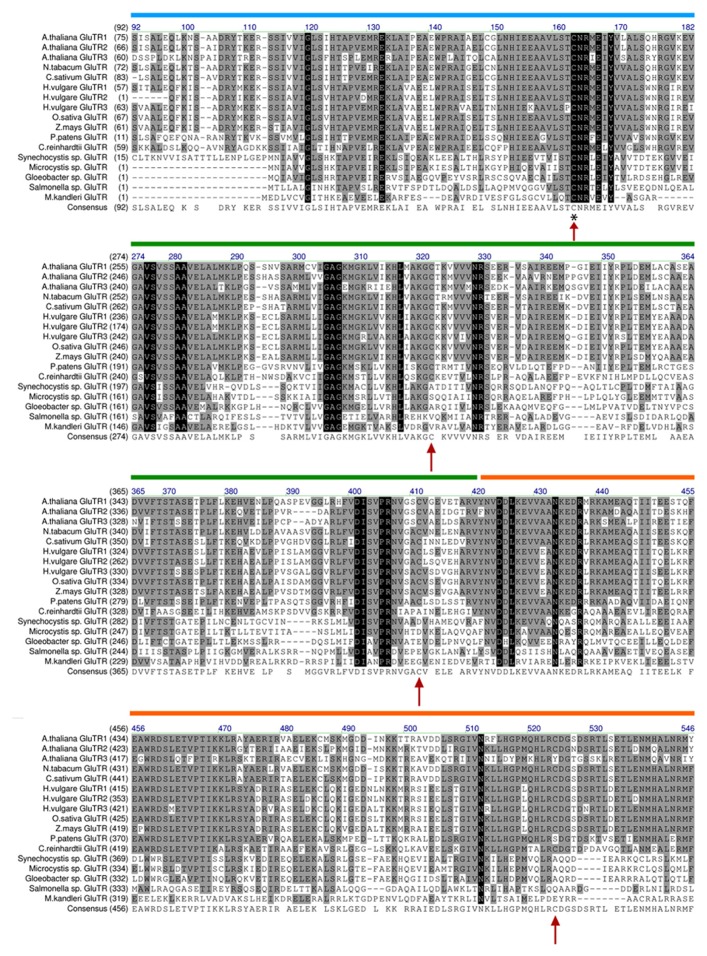
**Alignment of GluTR sequences of selected plant and bacterial species.** Arrows denote conserved cysteine residues in the GluTR sequence. The cysteine marked with an asterisk (*) was found to perform the nucleophilic attack on tRNA^GLU^ and is thus involved in the catalytic reaction of GluTR (see Moser et al., 1999/therein: Cys48 here: Cys163). Interestingly, only Cys163 is conserved in all analyzed GluTR amino acid sequences. The other cysteines (Cys322, Cys411, and Cys524) are only conserved in GluTR of higher plants (with the exception of *HEMA3* encoded GLUTR). The alignment is depicted without the proposed transit peptide of plant GluTRs and highlights the sequence harboring the conserved cysteines. Colored bars: domains annotated by Schubert et al. (2002). Blue: catalytic domain; green: NADPH-binding domain; orange: dimerization domain.

Based on biochemical analyses and the three-dimensional structure of the archaebacterial GluTR, the cysteine at position 48 (Moser et al., 1999,^2001^) nucleophilically attacks the aminoacyl bond of glutamyl-tRNA, leading to the formation of a thioester intermediate, which is subsequently reduced to GSA. This catalytically active cysteine is highly conserved in all GluTR molecules of non- and photosynthetically active organisms (Schubert et al., 2002 and **Figure 3**). Interestingly, GluTRs from higher plants (angiosperms) contain three additional conserved cysteine residues at position 322, 411, and 524 (with the exception of GluTR encoded by *HEMA3*; **Figure 3**). These highly conserved cysteines could be involved in the control of protein–protein interaction, stability, or activity.

**FIGURE 4:**
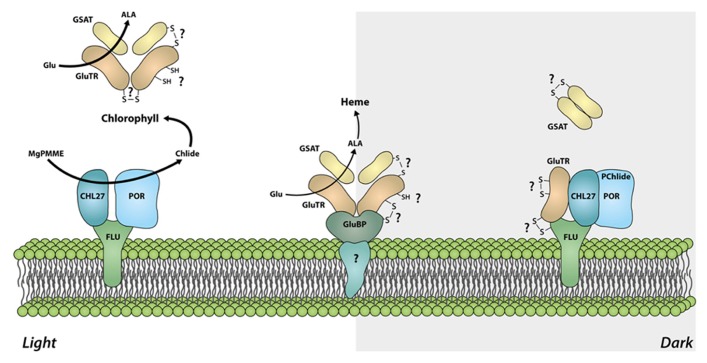
**Model of the redox-dependent organization of the ALA synthesizing enzyme complexes.** In light, the tetrapyrrole biosynthetic pathway is actively channeling substrates from GluTR to GSAT and ensures high activity of the ALA synthesizing enzymes and allocation of sufficient amounts of the precursors for the synthesis of different end products. In darkness, GluTR interacts with FLU, which is assembled with CHL27 and POR. This protein complex represses ALA synthesis. It is assumed that the reduction of target cysteine residues in intramolecular disulfide bonds of GluTR are the pre-condition for the formation of a heterotetrameric GluTR_2_GSAT_2_ complex or for high activity of ALA synthesizing enzymes in light. Oxidation of cysteine residues or formation of intra-/intermolecular disulfide bonds in darkness could additionally participate in the deactivation of GluTR (e.g., by stabilizing the interaction with FLU) and thus mediating the dark repression of ALA synthesis. Spatial separation of GluTR for heme synthesis by the GluTR-binding protein (GluBP) ensures ALA synthesis for the heme-synthesizing branch, although the bulk of ALA synthesizing activity is repressed by FLU.

First evidences for a functional significance of GluTR cysteine residues were obtained from analysis of an *Arabidopsis NTRC* knock out line. Although the *HEMA1* and *HEMA2 *transcript levels encoding GluTR1 and GluTR2 in the *ntrc *mutant are not changed compared to WT, the GluTR levels are significantly reduced. As GluTR evidently interact with NTRC, we conclude post-translational control of NTRC on the redox status of GluTR (Richter et al., 2013). It is suggested that a decreased capacity to reduce oxidized cysteine residues leads to destabilized GluTR. Alteration of the GluTR tertiary structure by oxidized cysteines or the formation of intramolecular disulfide bridges could serve as an explanation for the de-stabilization of oxidized GluTR, as it was reported for misfolded proteins which are easily susceptible to degradation (Betz, 1993; Hogg, 2003). One of the conserved cysteines (Cys524) is located in the dimerization domain (Schubert et al., 2002; **Figure 3**). Hence, it is not excluded that oxidation of cysteines interferes with the GluTR dimer formation. Then the thiol group of Cys524 in the dimerization domain has to be reduced as a pre-condition of GluTR homodimerization implicating that this process depends on the redox status of GluTR. As described above it is assumed that dimeric GluTR (GluTR_2_) forms a complex with dimeric GSAT (GSAT_2_) to facilitate substrate channeling between both enzymes. In terms of a potential redox regulation or a redox-dependent interaction of both GluTR_2_ and GSAT_2_ it is worth mentioning that GSAT also contains three highly conserved cysteine residues which are potential targets for thioredoxin-mediated reduction (Buchanan and Balmer, 2005). Future biochemical studies have to elucidate the function of these cysteines for the enzyme activity. But it is also not entirely excluded that interaction between GSAT_2_ and GluTR_2_ is mediated through conserved cysteine residues. A putative disulfide bridge between interacting GluTR and GSAT could be formed leading to the stabilization of the multimeric complex GluTR_2_GSAT_2_ (**Figure 4**). Formation of this complex would need a thiol group-reducing activity of enzymes, such as NTRC or thioredoxin. Both proteins are reported to interact with either GluTR or GSAT (Balmer et al., 2003; Richter et al., 2013).

As a key regulatory enzyme of tetrapyrrole biosynthesis, GluTR activity determines the flow rate of intermediates through the entire pathway (summarized in Czarnecki and Grimm, 2012). GluTR encoded by *HEMA1* was found to interact with the membrane-localized FLU protein which down-regulates GluTR activity in response to accumulating Pchlide, the substrate of the light-dependent POR (Meskauskiene et al., 2001; Meskauskiene and Apel, 2002; Richter et al., 2010). Kauss et al. (2012) described that GluTR forms a complex with CHL27 (subunit of the aerobic cyclase), POR, and FLU in darkness. This complex was not found in protein preparations from light-adapted plants. The authors proposed that GluTR is inactivated in darkness through binding to FLU, CHL27, and POR, whereas in light GluTR is released from this complex and becomes active. Hence, ALA synthesis undergoes a switch from “open” (active) in light to “closed” (inactive) in darkness to prevent accumulation of phototoxic intermediates of tetrapyrrole biosynthesis during dark periods. In principle, it is accepted that Pchlide-assembled POR in darkness is the cause for the rapid suppression of ALA synthesizing activity (Kauss et al., 2012). In consistency to the model Pchlide-mediated inactivation of GluTR, seedlings which do not accumulate Pchlide in darkness, do not show the FLU-mediated dark repression of ALA synthesis (Richter et al., 2010).

With regard to the physiological role of GluTR in the control of ALA biosynthesis, a plausible model for redox-dependent interaction of GluTR with other proteins in tetrapyrrole biosynthesis is presented (**Figure 4**). In light, an effective ALA formation is observed most likely due to channeling of substrates from GluTR to GSAT. Hence, high ALA synthesizing activity ensures a high metabolic flow rate in the entire tetrapyrrole biosynthetic pathways. In darkness GluTR interacts with the FLU-complex thereby repressing ALA synthesis and is perhaps released from the interconnection with GSAT (**Figure 4**). Although the above mentioned assumptions are rather suggestive and were not addressed in direct biochemical experiments yet, there are experimental indications for a light- and redox-dependent modification of GluTR interaction with these later enzymes of tetrapyrrole biosynthesis. Separation of soluble chloroplast protein fractions in blue-native gels revealed that GluTR assembles in high molecular weight protein complexes, including also the protein complex with FLU–POR–CHL27. These electrophoretic separations of protein complexes are performed under non-reducing conditions. When plastid protein extracts were separated under reducing conditions (or after treatment with reductants), at least part of GluTR assembled in the high molecular weight complexes are reduced and separated as monomer (Richter et al., 2013).

Moreover, GluTR is also attached to the thylakoid membrane by a GluTR-binding protein (GluBP; Czarnecki et al., 2011). The GluTR-binding protein attaches GluTR at the thylakoid membrane and spatially separates a small amount of GluTR from a soluble GluTR fraction. This fraction is suggested not to be accessible to the FLU-dependent inactivation in darkness (**Figure 4**). In terms of a putative redox-switch of GluTR between different complexes it is worth mentioning that the GluBP contains three cysteines which could stabilize the interaction of GluBP with GluTR under changing environmental conditions.

Additional studies are required to decipher the molecular processes of inactivation of bulk ALA synthesis in darkness. Among other essential amino acids in the tetratricopeptide repeat (TPR)-domain, a peptide motif mediating protein–protein interactions, FLU harbors also a conserved cysteine at the C-terminal end. The potential role of the cysteine residues of FLU and GluTR has to be explored for a potential redox-dependent shift of GluTR between different protein complexes during the diurnal light–dark transitions and other distinct environmental conditions in future. But a thiol-reducing mechanism for the FLU-dependent GluTR inactivation is plausible.

## REDOX CONTROL IN THE Mg-BRANCH

In this chapter we discuss the first reports on redox control in the Mg-branch of tetrapyrrole biosynthesis and emphasize potential thiol-based post-translational regulation of enzymes at these steps of tetrapyrrole biosynthesis. First evidence for a redox-dependent activity of MgCh were obtained, when Fuesler et al. (1984) reported on the sensitivity of MgCh activity from purified plastids to thiol-reacting reagents. Jensen et al. (2000) reported abolished ATPase activity of CHLI under oxidizing conditions and after blocking of conserved cysteines by *N*-ethylmaleimide (NEM). The block of cysteines did not affect the interaction between CHLI and CHLD. Therefore, the authors concluded that among four conserved cysteine residues at least one CHLI cysteine is essential for ATP binding and hydrolysis (Jensen et al., 2000). In the same study it was shown that activity of recombinant NEM-pretreated CHLH is lowered and presence of reducing reagents like DTT favors high MgCh activity. These results gave the first hints that thiol switches modulate activity of CHLI and the complete MgCh complex. Subsequently, the CHLI subunit was found to interact with thioredoxin(s) (Balmer et al., 2003). Further analysis confirmed a stimulatory function of thioredoxin on CHLI ATPase activity *in vitro *and *in vivo *(Ikegami et al., 2007; Luo et al., 2012). Thioredoxin is ferredoxin-dependently reduced suggesting that thioredoxin-mediated redox control of CHLI and the entire MgCh activity directs intermediates into the Mg-branch in response to activity of the photosynthetic apparatus (Luo et al., 2012). Light-dependent activation of MgCh ensures a high activity in day time and a rapid inactivation of MgCh during darkness. Together with the above-described post-translational repression of ALA synthesis (Meskauskiene et al., 2001), an additional inactivation of MgCh by a redox-dependent mechanism in darkness prevents the accumulation of phototoxic intermediates and ensures the bypass of Proto into the iron branch for heme synthesis. In light, thioredoxin accepts electrons from the photosynthesis-driven reduction of NADPH and ferredoxin, reduces disulfide bonds of CHLI and, thereby, activates the MgCh complex. Due to an immediate shift in the photosynthetic redox status in chloroplasts after the transfer from light to darkness, redox-sensitive thiol groups of cysteines in CHLI and CHLH would be accessible for oxidation which results in decreased activity of the MgCh. ALA feeding experiments in darkness indicates that the activity of MgCh and the successive enzymes within the Mg-branch is attenuated, but not dark-dependently blocked (Pontier et al., 2007; Richter et al., 2013). However, the dark-dependent repression of ALA synthesis is still believed to be the main point of action to prevent accumulation of Mg porphyrins in the dark.

Besides the thioredoxin-dependent post-translational activation of MgCh, additional experimental evidences provide insights into the redox-dependent control of enzymes of the Mg-branch. A thiol–disulfide exchange of cysteine residues of MgP methyltransferase was found to modulate CHLM stability and, consequently, its activity (Richter et al., 2013). The stimulatory effect of reduced cysteine residues on CHLM activity was confirmed in* in vitro* assays with recombinant CHLM and NTRC proteins. The *Arabidopsis ntrc* mutant possesses lower CHLM activity than the wild-type seedlings. These investigations conclusively demonstrate that reduction of the cysteines by redox regulators not only leads to stimulation of CHLM activity, but is also essential to maintain the structural integrity of the protein most likely in light and darkness. Further analysis will elucidate which of three highly conserved cysteine residues of CHLM are specific targets of NTRC-mediated redox control and which of these cysteines are essential either for the catalytic activity or the structural integrity of CHLM.

Previous analysis of NTRC suggested also another redox-related regulation of enzyme activities within the Mg-branch (Stenbaek and Jensen, 2010). As mentioned in chapter three NTRC interacts with 2-Cys PRX and provides electrons for the 2-Cys PRX-mediated detoxification of hydrogen peroxide (Perez-Ruiz et al., 2006). Stenbaek et al. (2008) addressed the sensitivity of the oxygen-consuming reaction of aerobic cyclase against ROS-mediated oxidation. They showed that the catalyzing detoxification of hydrogen peroxide by interaction of NTRC with 2-Cys PRX improves the *in vitro* enzymatic activity of the cyclase. This finding was supported by an earlier report of Bollivar and Beale (1996) showing increased cyclase activity in the presence of catalase. Very little is currently known about the catalytic mechanism and requirement of protein factors for the cyclase reaction. It cannot be excluded that the *in vitro* activity of the aerobic cyclase is inhibited by oxidation of the iron cluster, which is part of the complex catalytic oxidation reaction, resulting in the additional ring formation at MgPMME. It remains open if 2-Cys PRX participates in the *in vivo* reaction of cyclase. However, mutants with a knock down of PRXA (below 5% of WT level) and**a**parallel *PRXB* knock out do not show any alteration of Chl biosynthesis, although they accumulate high amounts of hydrogen peroxide in comparison to wild-type (Pulido et al., 2010). Future analysis of 2-Cys PRX mutants will help to elucidate the active contribution of 2-Cys PRX as ROS-detoxifying system in Chl biosynthesis.

## FINAL CONCLUSION

The thiol-based post-translational modulations serve in tetrapyrrole biosynthesis at multiple levels of oxidation and reductions. It is conceivable that disulfide–thiol exchange reaction affects activity, folding and stability of tightly controlled enzymes. Thus, these regulatory mechanisms contribute to synthesis of the matched amount of end products and facilitate a balanced flow of tetrapyrrole intermediates through the pathway without the risk of metabolite accumulations. It cannot be excluded that more target enzymes of oxidoreductase-mediated reactions will be found in tetrapyrrole biosynthesis. Moreover, the complete regulatory redox network of tetrapyrrole biosynthesis also includes the control of steady state levels of oxidants, which are easily generated in the highly light and oxygen-sensitive pathway of tetrapyrrole biosynthesis, and the supply and activation of a detoxification system against these oxidants. But, we have to admit that many processes are still poorly understood and the entire extent of the interplay between thioredoxins, glutaredoxins, and glutathionylations in tetrapyrrole metabolism cannot currently be envisioned. It remains a challenge to unravel the redox-dependent post-translational control and the redox signaling network of tetrapyrrole biosynthesis in the near future.

## Conflict of Interest Statement

The authors declare that the research was conducted in the absence of any commercial or financial relationships that could be construed as a potential conflict of interest.
